# Metastatic Pancreatic Cancer: Are We Making Progress in Treatment?

**DOI:** 10.1155/2012/898931

**Published:** 2012-12-04

**Authors:** Joanne Chiu, Thomas Yau

**Affiliations:** ^1^University Department of Medicine, Queen Mary Hospital, Hong Kong; ^2^University Department of Surgery, Queen Mary Hospital, New Clinical Building, 102 Pokfulam Road, Room 211B, Hong Kong; ^3^Centre for Cancer Research, The University of Hong Kong, Hong Kong

## Abstract

Development of systemic treatment for advanced pancreatic cancer (APC) has been challenging. After fluorouracil, gemcitabine (GEM) became the treatment of choice based on its benefit of symptom relief. Many cytotoxic agents have been combined with GEM in search of regimens with improved survival benefit. However, there were only marginal benefits in people with good performance status. Recently, the combination regimen consisting of oxaliplatin, irinotecan, fluorouracil, and leucovorin (FOLFIRINOX) was found to achieve unprecedented survival benefit and has become the preferred option for patients with good clinical conditions. On the other hand, many biological agents have been combined with GEM, but only erlotinib was found to derive statistically significant survival advantage. However, the effect was too small to be appreciated clinically. The effort in development of targeted therapy in APC continues. This paper summarized key findings in the development of chemotherapy and targeted therapy for APC patients and discussed future directions in management.

## 1. Introduction

Advanced pancreatic cancer (APC) is a dismal human cancer with median overall survival of merely 6 months. Approaches such as radiation, conventional chemotherapy, or combination of these modalities could not alter the disease course. Previously, fluorouracil (5FU) was given for treatment [1], but it was later taken over by gemcitabine (GEM) due to its effect on alleviation of disease-related symptoms [2]. However, the survival benefit of GEM was small. In desperate need of better treatment, much effort has been put on exploring new treatment regimes. It was once expected that biological agents could bring hope to this deadly cancer just like how they revolutionized the treatment of many other malignancies. When most of these agents seemed to fail, a nongemcitabine-containing combination chemotherapy (FOLFIRINOX) was found to give major improvement in response and survival and set the new standard of the treatment [3]. 

This paper summarized key development in the treatment of APC and discussed future possibilities in light of our current understanding. 

## 2. Traditional Chemotherapy

**Figure 1:**
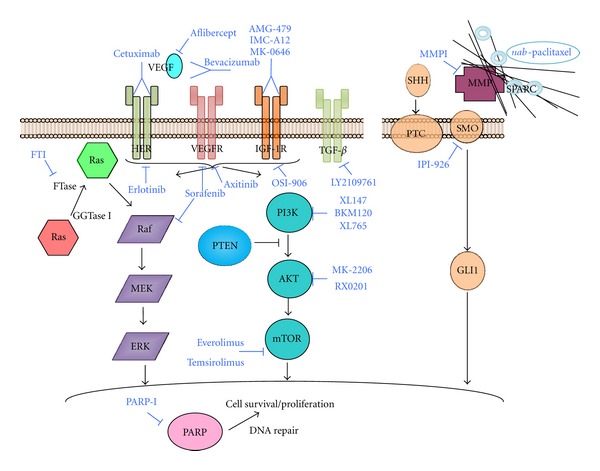
Selected signaling pathways and their targeting in advanced pancreatic cancer.

The progress in the development of systemic treatment of advanced pancreatic cancer (APC) has been slow (Figure 1). Traditionally, fluorouracil (5FU) was the only treatment option and provided only marginal benefit [1]. In 1997, Burris et al. in a randomized phase III trial showed that GEM was superior to 5FU in alleviation of disease-related symptoms and improving survival [2]. Although the improvement of median survival was merely 1.2 months (from 4.4 months with 5FU to 5.6 months with GEM, *P* = 0.0025), the 1-year survival rate was 18% for the GEM and 2% for the 5FU arms, respectively. GEM also gave a favorable clinical benefit rate (23.8% versus 4.8%, *P* = 0.0022). Thereafter, GEM took over to become the standard treatment of APC. 

Much effort was directed to improve the outcome of GEM treatment. GEM-based doublets with either platinum analogues or 5FU were extensively studied (Table 1). When GEM was combined with either cisplatin [4] or oxaliplatin [5] in individual trials, no benefits over single-agent GEM could be shown. Nevertheless, pooled analysis of these two European trials [4, 5] demonstrated that GEM-platinum doublet gave a 25% reduction in risk of progression (*P* = 0.0030) and 19% reduction in risk of death (*P* = 0.031) [6]. Yet, these benefits were mainly observed in asymptomatic patients with good performance status. On the other hand, addition of 5FU to GEM did not yield any survival improvement [7, 8]. This finding was further supported by trials where 5FU was substituted by the prodrug capecitabine (CAP) in the GEM doublet [9–11]. In these randomized studies, the GEM-CAP combination improved neither the progression-free survival (PFS), overall survival (OS), nor quality of life (Table 1). 

As APC is not the most prevalent cancer and most clinical trials contained around 200–300 subjects, it has been criticized that individual study has insufficient statistical power to detect the effect of combination chemotherapy. In view of this, a number of metaanalyses have been conducted (see Table 2). In summary, they primarily showed a significant benefit when GEM was combined with platinum or 5FU, but not with other cytotoxic agents. The maximum survival benefit of GEM-based combination chemotherapy was only marginal and mainly derived from patients with good performance status. Thus, the addition of platinum analogues to GEM was not recommended in general. 

Recently, in the French PRODIDGE 4/ACCORD 11 trial, the polychemotherapy regimen FOLFIRINOX (oxaliplatin, irinotecan, fluorouracil, and leucovorin) significantly improved the median OS of metastatic pancreatic cancer patients from 6.8 to 11.1 months when compared with single-agent GEM [3]. Such a magnitude of median OS improvement (4.3 months) was impressive but came with a price. Notably, 45% of the enrolled patients reported grade 3 or 4 adverse events (AEs). The most common AEs were diarrhea, fatigue, and vomiting. About 45% of patients experienced grade 3/4 neutropenia, including febrile neutropenia in 5.4%. In view of significant treatment-related toxicities, FOLFIRINOX is only recommended for patients with good performance status and medical conditions.

## 3. Biological Therapy in Pancreatic Cancer

Pancreatic cancer is a heterogeneous disease. Although the constitutively active K-ras^G12D^ allele mutation has been reported in 70%–90% of pancreatic cancers [15–17], no single oncogene addiction driving the growth of this cancer has been identified so far. According to the pancreatic cancer genome project, this cancer contains at least 63 genetic alterations on average. Besides K-ras, common mutations include FRAF (20%), AKT2 (20%), p16/CDKN2A (75%–80%), p53 (50%–75%), SMAD4 (50%–60%), and BRCA2 (10%) [17]. A number of cellular signaling pathways have been implicated in the pathogenesis and maintenance of this cancer, including hedgehog signaling, K-ras signaling, and transforming growth factor beta (TGF-*β*) signaling, to name a few. The following sections will discuss the key findings in the quest of better treatment of APC using biological therapy.

### 3.1. Ras Signaling

As K-ras mutation is the commonest mutation in pancreatic cancer, it was one of the earliest targets for drug development. The functional sites of this protein are difficult to access, so scientists have attempted to target the enzyme taking part in modification/activation of Ras called farnesyltransferase. Examples of farnesyltransferase inhibitors (FTIs) include tipifarnib and SCH66336. These agents as single agents or when combined with GEM showed no appreciable activity in clinical trials [18–21], and the failure of FTIs was suspected to be due to recruitment of alternative pathways for Ras activation. Nowadays, people are still developing novel methods such as antisense and RNA interference and inhibitors of its key effector MEK kinase to target the Ras protein or its signaling.

### 3.2. Epidermal Growth Factor Receptor (EGFR)

EGFR is overexpressed in human pancreatic cancer and is suspected to play an important role in metastasis [22]. Both small molecule tyrosine kinase inhibitors (TKIs) and monoclonal antibodies are well-known strategies in targeting the EGFRs. Erlotinib is a TKI of this class and has gained attention in the treatment of APC. In the PA.3 study, which is a multicentre, randomized, double-blind, and placebo-controlled clinical study of erlotinib in combination with GEM in APC, the erlotinib-GEM combination compared with single-agent GEM showed a statistically improvement in PFS (HR 0.77 (95% CI, 0.64 to 0.92; *P* = 0.004)) and 1-year survival (23% versus 17%; *P* = 0.023) [23]. The median OS was also increased, but the improvement was small (6.24 versus 5.91 months, HR 0.82 (95% CI, 0.69 to 0.99; *P* = 0.038)). This combination is not considered a clinically meaningful option or a cost-effective choice by many physicians.

Cetuximab is a chimeric monoclonal antibody against ErbB-1 receptors with high specificity. 

In a phase II study combining cetuximab and GEM in untreated APC patients, there appeared to be encouraging results [24]. But this combination did not show superiority to single-agent GEM when tested in a randomized phase III study (6.3 versus 5.9 months, HR 1.06; (95% CI, 0.91 to 1.23; *P* = 0.23)) [25]. In order to “intensify” the anti-EGFR activity, people have attempted to combine another anti-EGFR antibody, panitumumab, with erlotinib and GEM [26]. Nevertheless, excessive toxicities shown in the phase II study precluded further pursuit using this combination. These research studies suggest that EGFR expression, though correlating with tumor aggressiveness, does not necessarily predict response to anti-EGFR therapy. The role of this class of treatment remains to be defined.

### 3.3. Angiogenesis

Tumor growth is sustained by angiogenesis. Vascular endothelial growth factor (VEGF) expression was found to be associated with liver metastasis and poor prognosis in pancreatic cancer [27]. However, trials on anti-VEGF therapy in APC have been disappointing. 

Bevacizumab is the most widely used anti-VEGF antibody and has been shown to enhance the effect of chemotherapy in many other cancers. In the Cancer and Leukemia Group B (CALGB) 80303 trial, patients with APC were randomized to receive GEM with or without bevacizumab [28]. The addition of bevacizumab, however, did not extend overall survival. Similarly, in the phase III AVITA trial, patients were given GEM with erlotinib plus either bevacizumab or placebo. Despite improved PFS (median: 4.6 months versus 3.6 months, HR 0.73, *P* = 0.0002), it did not translate into improved overall survival [29]. Attempts of other oral inhibitors of VEGF receptor, such as axitinib [30] or aflibercept [31], or multikinase inhibitor like sorafenib [32], also failed to improve survival. 

### 3.4. Other Potential Treatment Strategies in the Near Future

The chemotherapy-resistant nature of APC and failure of antiangiogenesis therapy have prompted revision on the existing model of tumor microarchitecture. Increasing evidence suggests that pancreatic cancer is characterized by “tumor desmoplasia,” the presence of dense stromal tissue with decreased vascular density that penetrates and envelopes the tumor [33]. It is now believed that this stromal tissue impairs drug delivery, leading to treatment resistance. *nab*-paclitaxel is an albumin-bound formulation of paclitaxel with enhanced affinity to a stromal protein called secreted protein acidic and rich in cysteine (SPARC), which is overexpressed in the stroma of APC. As a result, *nab*-paclitaxel is concentrated around the tumor. Early phase I/II trial demonstrated promising result when *nab*-paclitaxel was combined with GEM in untreated APC patients. The reported response rate was 48%, 1-year overall survival rate was 48%, and the median OS was 12.2 months [34]. Phase III trial of this combination is underway. Novel technological advance such as nanotechnology might help develop drug therapy that can overcome the barrier imposed by the dense stromal microenvironment. Other ongoing pieces of research aiming at manipulating the signaling or function of microenvironment include hedgehog pathway inhibitors and hyaluronidase which breaks down the hyaluronan in the extracellular matrix. 

Pancreatic cancer is a complicated disease. Increasing understanding of the molecular pathways to pathogenesis and growth of pancreatic cancer showed that a number of other signaling pathways are also implicated. For instance, insulin-like growth factor receptor (IGFR-1) mRNA in pancreatic cancer was more than 30 times that in normal pancreatic tissue, and abnormal regulation of IGF-1 autocrine loop was associated with increased tumorigenicity [35, 36]. Another signaling pathway, transforming growth factor beta (TGF-*β*), has both the effects of regulation of cell growth and mediation of cancer cell proliferation and metastasis [37]. Drug development that targets these pathways is still preliminary. 

In the era of personalized medicine, there is an unmet need for development of biomarkers to guide management. Carbohydrate antigen 19.9 (CA 19.9) is a widely accepted surrogate marker for treatment response, although it has only modest sensitivity and specificity [38]. Furthermore, K-ras wild type status appeared to derive better survival from erlotinib than K-ras mutant tumor, and so did patients who developed rash during erlotinib treatment. Pharmacogenetics also plays a role. Depressed level of GEM metabolism gene products such as deoxycytidine kinase (dCK) and ribonucleoside reductases M1 and M2 (RRM1, RRM2) has been correlated with treatment resistance to GEM [39–41]. Low expressers of the nucleoside transporter-1 (hENT1) reduced uptake of GEM in cell and were found to have poorer prognosis [40]. In the future, we wish to identify biomarker that can predict response of treatment and stratify patients accordingly to enhance the treatment effect.

## 4. Conclusion

The development of treatment for APC has been challenging. Although many biological agents were tested, recent advances in clinically significant treatment are dominated by chemotherapy. The FOLFIRINOX regimen has gained increased acceptance, and the effect of *nab*-paclitaxel-GEM combination is awaited from phase III study. The future prospective depends on further understanding in the tumor biology, targeting various growth factor signaling pathways, and development of new technologies, including identification of biomarkers that predict treatment response.

## Figures and Tables

**Table 1:** Major phase III GEM-based combination chemotherapy.

Regime	No. pts	ORR (%)	PFS/TTP (mo)	OS (mo)	Reference
GEM + cisplatin	195	10.2	5.3	7.5	Heinemann et al. [4]
GEM		8.2	3.1	6.0	
GEM + oxaliplatin	832	(*P* = 0.11)	2.7	5.7	Poplin et al. [5]
GEM			2.6	4.9	
GEM + 5FU	322	6.9	3.4*	6.7	Berlin et al. [7]
GEM		5.6	2.2	5.4	*P = 0.022
GEM + capecitabine	533	19.2	5.3	7.1	Cunningham et al. [9]
GEM		12.4	3.8	6.2	
GEM + capecitabine GEM	319	QoL same	n/a	n/a	Bernhard et al. [10]
GEM + capecitabine	319	10.0	4.3	8.4 (10.1*)	Herrmann et al. [11]
GEM		7.8	3.9	7.2 (7.4)	(*KPS 90–100; *P* = 0.014)

Abbreviation: ORR: overall response rate; PFS: progression free survival; TTP: time to progression; OS: overall survival; QoL: quality of life.

**Table 2:** Selected metaanalysis of GEM-based combination chemotherapy.

Reference	No. of pts	HR (all)	HR for platinum	HR for 5FU/capecitabine	HR for other agents
Heinemann et al. [12]	4465	0.91*	0.85	0.9	0.99
Sultana et al. [13]	9970	0.91	—	—	—
Vaccaro et al. [14]	2422	0.87	0.94 (cisplatin, *P* = 0.61)	0.86 (*P* = 0.04)	—
			0.86 (oxaliplatin, *P* = 0.04)		

*ECOG PS 0-1: HR 0.76, *P* < 0.001; ECOG PS 2: HR 1.08, *P* = 0.40.

Abbreviations: HR: hazard ratio; 5FU: fluorouracil.

## Abbreviations

FTI: Farnesyltransferase inhibitor
GGTase I: Geranylgeranyltransferase I
IGF-1R: Insulin-like growth factor 1 receptor
HER: Human epidermal growth factor receptor
MEK: Mitogen-activated protein kinase/ERK kinase
MMP: Matrix metalloproteinases
PTC: Patched-1 receptor
PTEN: Phosphatase and tensin homologue deleted on chromosome ten
SHH: Sonic hedgehog
SMO: Smoothened
SPARC: Secreted protein acidic and rich in cysteine
TGF-*β*: Transforming growth factor *β*
VEGF: Vascular endothelial growth factor
VEGFR: VEGF receptor.
